# Studying the therapeutic effects of hemoperfusion with continuous venovenous hemofiltration in paraquat-poisoned patients by the ratio of residual normal lung in 3D-CT image

**DOI:** 10.1186/1471-227X-12-S1-A1

**Published:** 2012-12-18

**Authors:** Liu Peng, Zhang Hong-tao, Liang Yu-guang, He Jin, Li Yue-qi, Li Gong-jie, Zhou Yue-su

**Affiliations:** 1 Department of Emergency, 302 Hospital of PLA, Beijing 100039, China; 2 Department of Radiology, Affiliated hospital of Academy of Military Medical Sciences, Beijing 100071, China; 3 Department of Pharmacology, Academy of Military Medical Sciences, Beijing 100071, China

## Objective

Paraquat poisoning(PQ) by ingestion is often fatal and is a significant public health problem worldwide.The lung is the major target organ for PQ poisoning. The study is aimed to investigate the ratio of residual normal lung in 3D-CT image in evaluating the therapic effects of continuous venovenous hemofiltration(CVVH).

## Methods

Nighty-five patients with acute paraquat poisoning were randomly divided into hemoperfusion(HP) group (46 cases) and HP-CVVH group (49 cases). The mortality, survival duration and the ratio of residual normal lung in 3D-CT image between the two groups were compared and analyzed.

## Results

There were no significant differences in mortality (28.26% versus 24.49%) between the two groups on day 28 after poisoning. The mean time between poisoning and death in HP-CVVH group was (5.2±2.1) days, which was significantly longer than that (3.8±1.7) days in HP group (P<0.05). The ratio of residual normal lung in 3D-CT image on 6th day after poisoning in HP-CVVH group was (31.80±12.71)%,which was significantly higher than that (25.60±14.06)% in HP group (P < 0.05).

## Conclusion

The combined therapy of HP and CVVH could prevent advances in lung injury induced by acute paraquat poisoning and prolong survival time, but failed to reduce mortality of paraquat-poisoned patients.

